# Beneficial Immunomodulatory Effects of Fluticasone Propionate in *Chlamydia pneumoniae*-Infected Mice

**DOI:** 10.3390/pathogens10030338

**Published:** 2021-03-14

**Authors:** Dóra Paróczai, Anita Sejben, Dávid Kókai, Dezső P. Virok, Valéria Endrész, Katalin Burián

**Affiliations:** 1 Department of Pulmonology, University of Szeged, Alkotmány str. 36., 6772 Deszk, Hungary; 2 Department of Medical Microbiology and Immunobiology, University of Szeged, Dóm sqr. 10., 6720 Szeged, Hungary; kokai.david@med.u-szeged.hu (D.K.); virok.dezso.peter@med.u-szeged.hu (D.P.V.); endresz.valeria@med.u-szeged.hu (V.E.); burian.katalin@med.u-szeged.hu (K.B.); 3 Department of Pathology, University of Szeged, Állomás str. 1., 6720 Szeged, Hungary; sejben.anita@med.u-szeged.hu

**Keywords:** inhaled corticosteroid, fluticasone, *Chlamydia pneumoniae*, respiratory infection, chemokine, interferon

## Abstract

The associations between inhaled corticosteroid (ICS) use and pulmonary infections remains controversial. *Chlamydia pneumoniae (C. pneumoniae)* accounts for asthma exacerbations; however, there are no data regarding ICS effects on *C. pneumoniae* infections. Thus, we investigated whether fluticasone propionate (FP) or budesonide (BUD) could affect *C. pneumoniae* infection in vitro and in vivo, focusing on the possible mechanisms that lead to potential anti-chlamydial outcomes. We performed direct qPCR to detect *C. pneumoniae* growth in infected, FP-treated, and BUD-treated A549 cells. Furthermore, FP or BUD was administered by inhalation to *C. pneumoniae*-infected mice. The recoverable *C. pneumoniae* was determined by indirect immunofluorescence. Expression levels of interferon (IFN)-γ and IFN-γ inducible chemokines were assessed by qPCR. We measured the protein concentrations of IFN-γ and of other cytokines that potentially participate in the anti-chlamydial response by ELISA. We found that FP treatment suppressed *Chlamydia* growth in A549 cells and in mice. Higher levels of IFN-γ gene expression were observed in FP-treated mice compared to the untreated and BUD-treated mice (*p* < 0.0001). IFN-γ and anti-chlamydial protein MIG/CXCL9 values were significantly higher after FP inhalation. Collectively, FP, but not BUD, suppressed *C. pneumoniae* growth *in vitro* and *in vivo*, which was likely due to the enhanced IFN-γ related responses.

## 1. Introduction

Inhaled corticosteroids (ICSs) are regarded as the most effective treatment for asthma and chronic obstructive pulmonary disease (COPD) to reduce the risk of exacerbation and improve lung function; however, ICSs have been associated with increased risk of pneumonia [1]. Earlier studies have provided controversial data about the potential risk of pneumonia in patients using ICSs and emphasise the differences in their mechanisms of action [2,3]. It is well established that budesonide (BUD) and fluticasone propionate (FP) show differences in their pharmacokinetic, physicochemical, and even in immunosuppressive properties, which can explain their distinct effects on respiratory infections and exacerbations [4,5]. As ICSs may have anti-inflammatory effects on the outcomes of respiratory infections, BUD and FP were studied to determine whether they can affect common viral or bacterial infections associated with COPD and asthma exacerbations. Previous studies have revealed that BUD can inhibit rhinovirus replication and beneficially modulate cytokine responses in vitro depending on the type of infected cell [6,7]. Although both BUD and FP can suppress pro-inflammatory cytokine expression, BUD had a greater impact on antimicrobial proteins [8]. Furthermore, BUD came into focus during the SARS-CoV-2 pandemic, and it was examined in coronavirus HCoV-229E infection, where it was found to decrease the expression of the viral entry receptor and infection-induced cytokines, especially interleukin (IL)-6, IL-8, and interferon (IFN)-γ, resulting in inhibited viral replication in vitro [9]. The transcription of genes involved in cytokine and chemokine responses, particularly CCL-5, also known as RANTES (regulated on activation, normal T cell expressed and secreted); and nuclear factor κB (NF-κB)-dependent gene expression, is altered more extensively by FP than by BUD [10].

However, it remains unclear why ICSs have different effects on the immune responses to respiratory infections. FP has been reported to cause a ten-fold more potent inhibitory effect on airway immune cells and pro-inflammatory cytokine production due to its prolonged presence in the airway mucus, as compared to BUD [11,12]. Since BUD is in conjugated form intracellularly and creates a depot before it is activated [13], its effect is still unexplained in intracellular bacterial infections. In contrast to in vitro studies, BUD attenuated pulmonary antibacterial host defence and increased the number of viable bacteria in mouse lungs [14].

*Chlamydia pneumoniae* (*C. pneumoniae*) is an obligate intracellular bacterium that propagates in respiratory epithelial cells, is responsible for community-acquired atypical pneumonia, bronchitis, pharyngitis, sinusitis, and is implicated in the development of severe asthma and acute exacerbations [15,16]. We have previously revealed that a former *C. pneumoniae* infection was associated with altered cytokine responses in patients with asthma [17]. However, to our knowledge, the consequences of ICS use on *C. pneumoniae* infection have not been investigated yet. To address the question of whether ICS use could directly or indirectly influence *C. pneumoniae* infection, we investigated the effects of FP and BUD treatment in an infected mouse model. We hypothesised that *C. pneumoniae* replication and infection-induced immune responses, especially anti-chlamydial IFN-γ, IFN-related chemokine production, and IFN-γ triggered gene expressions, could be influenced by the administration of ICSs. Since BUD and FP have different immunomodulatory effects, they can cause distinct alterations in the immune response to *C. pneumoniae* infection. To investigate this, we assessed the effects of FP and BUD on the in vitro and in vivo growth of *C. pneumoniae* in airway epithelial cells and in mice.

## 2. Results

### 2.1. FP Suppressed C. pneumoniae Replication in A549 Cells

**Figure 1:**
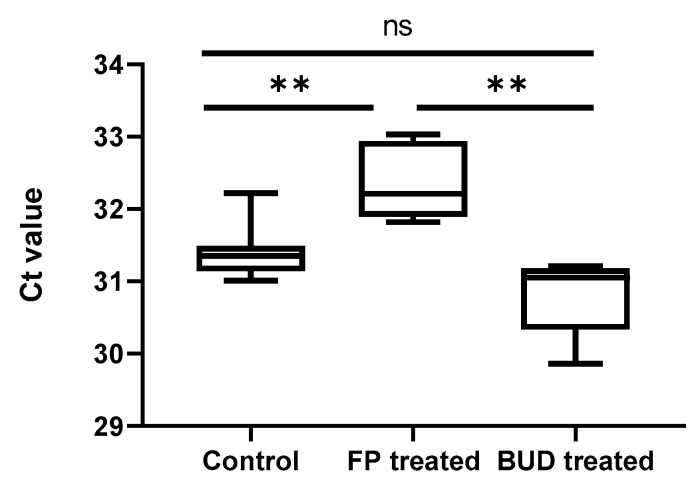
*C. pneumoniae* growth in A549 cells treated with budesonide (BUD) or fluticasone propionate (FP). A549 cells were treated with BUD or FP for 24 h before, and for 48 h after infection with *C. pneumoniae* (0.01 MOI). Growth of *C. pneumoniae* was estimated by direct qPCR, as described in the Materials and Methods section. The concentration of *C. pneumoniae* is shown in terms of Ct value. Error bars denote the mean ±SD of five parallel cultures. Asterisks indicate significant differences, ** *p* < 0.01; ns means not significant difference.

We tested *C. pneumoniae*-infected A549 cells to investigate whether FP or BUD treatment could modulate bacterial growth. As shown earlier, assessment of chlamydial genome concentration by direct qPCR correlated with manual fluorescent microscopic quantitation, wherein qPCR was used to measure the concentration of *Chlamydia* in infected cells [18]. We assessed *C. pneumoniae* growth in FP- and BUD-treated epithelial cells based on the cycle threshold (Ct) values. FP treatment resulted in significantly higher Ct values, indicating suppressed *C. pneumoniae* growth, compared to that measured in BUD-treated (32.35 ± 0.51 vs. 30.81 ± 0.55, *p* < 0.01) and untreated control (32.35 ± 0.51 vs. 31.41 ± 0.39, *p* < 0.01) cells. BUD treatment did not affect *C. pneumoniae* growth significantly (Figure 1). Given that our *in vitro* observations could have significant clinical relevance, we next addressed the elucidation of possible immunomodulating effects of FP and BUD in mice to explore the underlying mechanisms.

### 2.2. FP Inhibited C. pneumoniae Growth in the Lungs of Mice

**Figure 2:**
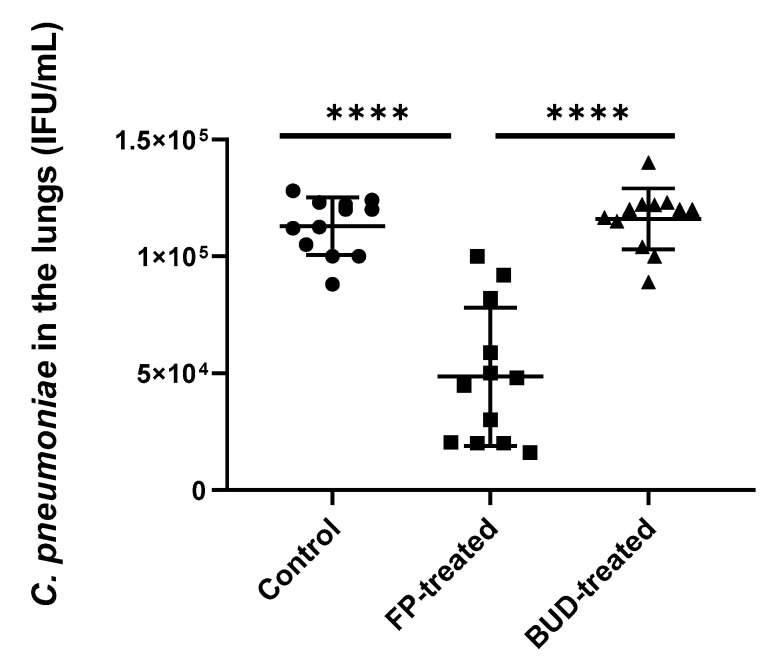
Quantity of recoverable *C. pneumoniae* in mouse lungs. Supernatants of lung homogenates were cultured on McCoy cells, and the number of recoverable *C. pneumoniae* was counted by indirect immunofluorescence test after two days of incubation. Symbols show data from individual mice. Horizontal lines indicate mean ± SD. Significant differences are indicated by asterisks, **** *p* < 0.0001.

We found that the viable number of *C. pneumoniae* was significantly lower in FP-treated mice compared to the control group (5.33 × 10^4^ ± 3.42 × 10^4^ inclusion-forming unit (IFU)/mL vs. 1.13 × 10^5^ ± 1.28 × 10^5^ IFU/mL, *p* < 0.0001) (Figure 2). A similar trend was observed upon comparing FP-treated mice with BUD-treated mice (5.33 × 10^4^ ± 3.42 × 10^4^ IFU/mL vs. 1.15 × 10^5^ ± 1.42 × 10^4^ IFU/mL, *p* < 0.001). In contrast, no inhibition was detected in BUD-treated mice compared to the control (Figure 2).

### 2.3. Effects of FP and BUD on Chlamydia-Infected Lung Tissue Histopathology

**Figure 3:**
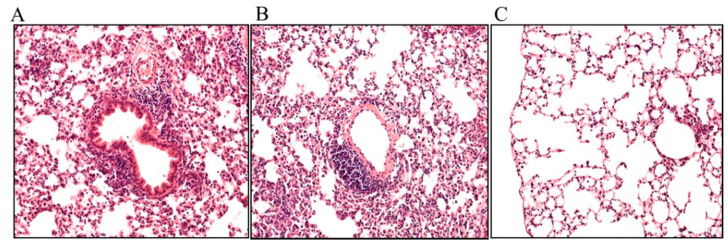
The effects of inhaled corticosteroids (ICSs) on *Chlamydia*-induced histopathology in BALB/c mouse lung tissues. Representative HE-stained sections of *C. pneumoniae*-infected, untreated (**A**), *C. pneumoniae*-infected, BUD-treated (**B**), and *C. pneumoniae*-infected, FP treated (**C**) lung tissues (magnification 200×).

In the haematoxylin–eosin (HE)-stained mouse lung tissues, a distinctive difference in the general blueish appearance of the background in the control and BUD-treated specimen was observed, which was caused by extensive lymphoid infiltration. Even though the inflammation appeared diffusely, accentuated perivascular and peribronchial infiltration was also observed at higher magnification (Figure 3A,B). Similarly, in the FP-treated mouse lung tissues, lymphocytic and plasmacytic infiltration was observed; centriacinar emphysema was also visible, with thin alveolar septa (Figure 3C).

### 2.4. Effects of FP and BUD Treatment on Gene Expressions Related to IFN-γ and Corticosteroid Responses in C. pneumoniae Infected Mice

**Figure 4:**
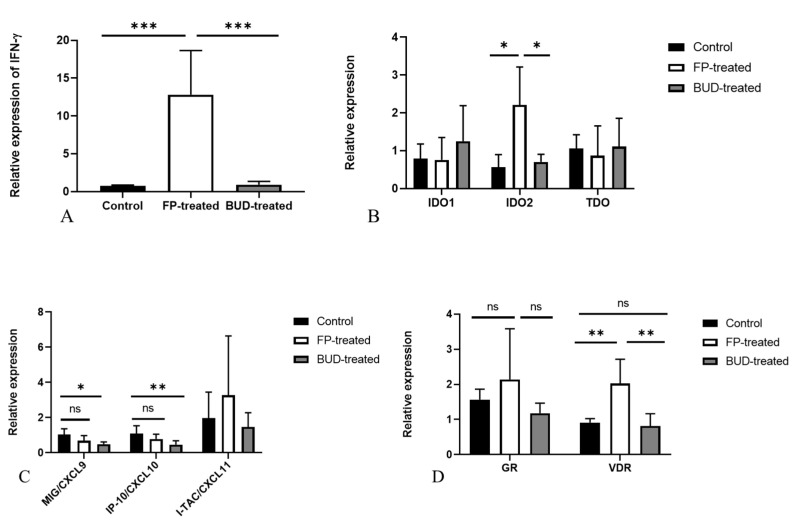
Relative gene expressions in *C. pneumoniae*-infected and treated mouse lungs. Gene expression of IFN-γ (**A**), IDO1, IDO2, TDO (**B**), IFN-γ-inducible chemokines (**C**), VDR, and GR (**D**) determined by qPCR. Relative expression was normalised to β-actin gene expression and calculated by the 2^−(ΔΔCt)^ method. Significant differences are indicated with asterisks, * *p* < 0.05, ** *p* < 0.01, *** *p* < 0.001; ns means not significant difference.

Next, we explored whether the expression of IFN-γ and IFN-γ induced genes was altered in FP- and BUD-treated lung tissues, including the typical, inducible defence genes against *Chlamydia* infection, such as indoleamine 2,3-dioxygenase 1 (IDO1), IDO2, MIG/CXCL9, IP-10/CXCL10 and I-TAC/CXCL11. qPCR using total RNA isolated from homogenised lung tissues revealed that the relative expression of IFN-γ was significantly enhanced in FP-treated mice (*p* < 0.001) compared to BUD-treated and control mice, the relative expression was 12.8 ± 5.8 vs. 0.9 ± 0.43 and 12.8 ± 5.8 vs. 0.75 ± 0.1, respectively (Figure 4A).

Our previous studies showed that the IFN-γ inducible IDO1 and IDO2 are highly expressed in *C. pneumoniae*-infected mouse lungs [19]. Thus, we next investigated the effects of FP and BUD treatment on the expression of IFN-γ inducible IDO1, IDO2, and tryptophan 2,3-dioxygenase (TDO) involved in the metabolism of amino acid tryptophan that is essential for *Chlamydia* growth. Our results indicated a significantly increased IDO2 expression in the FP-treated mice compared to the control and BUD-treated group (*p* < 0.05). This phenomenon was not observed in IDO1 and TDO expression (Figure 4B).

To test the consequence of increased IFN-γ release, we determined the relative expression levels of IFN-γ related chemokines. Unexpectedly, we found that BUD significantly decreased the expression of MIG/CXCL9 (*p* < 0.05) and IP-10/CXCL10 (*p* < 0.01) compared to untreated *C. pneumoniae* infected control mice. However, the relative expression of MIG/CXCL9 and IP-10/CXCL10 was not altered significantly in FP-treated mice. I-TAC/CXCL11 showed similar expression levels in all groups (Figure 4C).

Furthermore, we investigated the expression of glucocorticoid receptor (GR) and Vitamin D receptor (VDR) genes, as both receptors perform immunomodulatory functions [20,21]. Interestingly, our results demonstrated that FP treatment increased VDR expression significantly compared to control and BUD-treated mice (*p* < 0.01), whereas BUD treatment did not affect VDR expression. We found no statistically significant difference in GR expressions in *C. pneumoniae*-infected BUD- and FP-treated mice (Figure 4D).

### 2.5. Anti-Chlamydial IFN-γ and MIG/CXCL9 Protein Production Are Enhanced by FP Treatment

**Figure 5:**
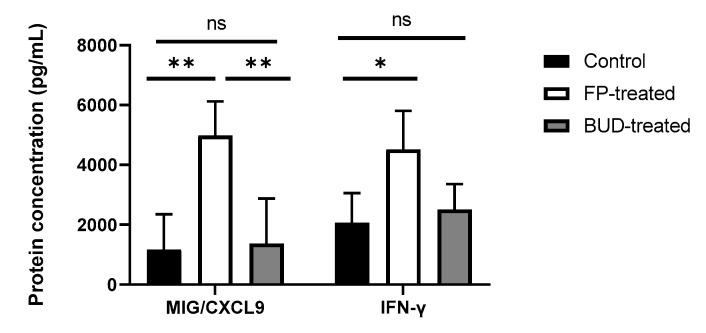
Effect of ICSs on IFN-γ and MIG/CXCL9 production in *C. pneumoniae*-infected mouse lungs. IFN-γ and MIG/CXCL9 protein concentrations were determined by ELISA in the supernatants of homogenised lung tissues of infected FP-treated, BUD-treated, and untreated mice. Data are shown as pg/mL. Bars denote mean ± SD of each group (n = 12). Significant differences are indicated with asterisks, * *p* < 0.05, ** *p* < 0.01; ns means not significant difference.

As previously described, IFN-γ exhibits crucial anti-chlamydial activity by inducing chemokine production and increasing anti-chlamydial gene expression [22]. To determine whether ICSs altered gene expression, and influenced the levels of IFN-γ and related chemokines, we estimated IFN-γ and MIG/CXCL-9 protein concentrations in the supernatants of lung homogenates for *C. pneumoniae*-infected mice (Figure 5). According to our results, IFN-γ production was significantly increased after FP treatment compared to untreated infected control (4519.77 ± 1289.08 pg/mL vs. 2060.07 ± 995.76 pg/mL, *p* < 0.05). BUD treatment did not affect IFN-γ production significantly compared to FP-treated or control mice.

We examined whether MIG/CXCL9 production at the protein level was changed in association with alterations in IFN-γ production in vivo. We found that FP-treated mice showed a higher protein level of MIG/CXCL9, compared to that in untreated controls (4984 ± 1137 pg/mL vs. 1169 ± 1178 pg/mL, *p* < 0.01). Furthermore, a significant increment in the level of MIG/CXCL9 was detected in FP-treated lung tissues compared to the BUD-treated mouse lungs (4984 ± 1137 pg/mL vs. 1370 ± 1509 pg/mL, *p* < 0.01) (Figure 5).

### 2.6. Effects of BUD and FP Treatment on the Secretion of Th17 and Th2 Cytokines in C. pneumoniae-Infected Lung Tissues

**Figure 6:**
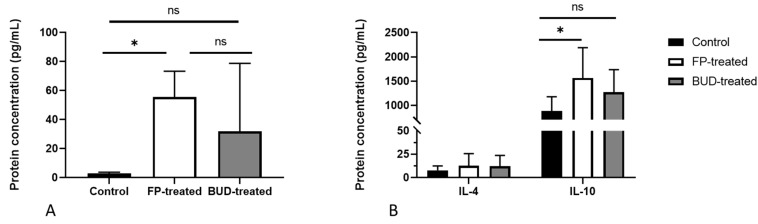
Effects of BUD and FP on the secretion of interleukin (IL)-17A, IL-4 and IL-10 in control and treated mice. IL-17A (**A**), IL-4, and IL-10 (**B**) protein concentrations were determined by ELISA in the supernatants of homogenised lung tissues of infected FP-treated, BUD-treated, and untreated mice. Bars denote mean± SD of each group (n = 12). * *p* < 0.05, ns means not significant difference.

ELISA of supernatants from control and treated lung samples was performed to elucidate the influence of ICS treatment on cytokine production. As previously reported by us, IL-17A has an indirect anti-chlamydial activity in vivo [23]. Thus, we investigated the impact of ICSs on IL-17A production in infected mouse lungs (Figure 6A). We found a significantly elevated IL-17A level in FP-treated mouse lungs compared to the control group (55.59 ± 17.7 pg/mL vs. 3.06 ± 0.67 pg/mL, *p* < 0.01). Although increased IL-17A production was observed in BUD-treated mice, it did not differ significantly from the control and FP- treated mice.

As Th2 cytokines play a pivotal role in modulating lung inflammation, we evaluated whether FP and BUD could affect IL-4 and IL-10 secretion in *C. pneumoniae*-infected mice (Figure 6B). Our results indicated unaltered IL-4 production, whereas IL-10 levels changed in a different manner. We detected a significantly higher amount of IL-10 in FP-treated lung tissues, but not in the BUD-treated lungs, in comparison with the untreated infected mice (1757.28 ± 602.93 pg/mL vs. 739.67 ± 19.70 pg/mL, *p* < 0.05).

## 3. Discussion

ICSs are widely used to treat COPD and asthma, as they show a broad range of anti-inflammatory properties and improve lung function. However, the association between ICS use and the risk of pneumonia remains unclear, as different ICSs have distinct effects on infections. Several studies have been conducted to reveal their possible connection to respiratory infections; nevertheless, clinical data tend to be contradictory [2,24,25]. ICSs have different pharmacological and immunomodulatory effects considering responses to viral infections, immune cell functions, and cytokine production [4]. As exacerbations of obstructive lung diseases are mainly caused by viral or bacterial infections, ICSs were investigated from a new perspective, and their effects on antimicrobial defence were examined.

FP and BUD affected pro-inflammatory epithelial responses, and they presented antiviral and anti-inflammatory activities differentially. In vitro studies have shown that BUD is effective in counteracting rhinovirus replication, inhibiting cytokine production, and enhancing antimicrobial protein secretion; however, in vivo, it suppressed pulmonary host defence genes that are essential for bacterial clearance [6,14,26,27]. Moreover, inhalation of BUD has been shown to lead to significant alterations in the regulation of anti- and pro-inflammatory genes involved in eliminating respiratory pathogens [28].

FP is regarded as a more potent inhibitor of pro-inflammatory genes and cytokines, although it can preserve the production of several cytokines, especially IFN-γ, to reverse its exaggerated effect on Th2 response in allergic inflammation. Inhibitory effects of this topically active ICS are not well-established in respiratory infections, despite the fact that FP is far more potent in cytokine production than other ICSs [29].

*C. pneumoniae* is implicated in asthma exacerbations, and there is increasing evidence indicating that it plays a role in asthma pathogenesis, leading to altered cytokine responses, decreased lung function, and heightened disease severity [30,31,32]. Thus, we aimed to investigate whether ICSs could have beneficial modulatory effects on *Chlamydia* infection in vitro and in vivo, and thus, define the possible underlying mechanisms.

Collectively, our reported data inspire several exciting concepts that could have practical outcomes for respiratory physicians. Our in vitro results suggest that *C. pneumoniae* growth is suppressed in infected epithelial cells by FP but not by BUD. Thus, we tested our hypothesis whether FP could inhibit the growth of *C. pneumoniae* in vivo. We found that the number of recoverable *C. pneumoniae* decreased in *C. pneumoniae*-infected mouse lungs after FP treatment, as observed in A549 epithelial cells. It is well established that the IFN-γ is the main regulator of *Chlamydia* elimination and can trigger various mechanism leading to Chlamydia inhibition. IFN-γ exposure induced IDO activity, which is responsible for tryptophan degradation. As *Chlamydiae* are tryptophan auxotrophs, increased IDO activity results in a restricted *Chlamydia* growth in vitro and in vivo [19,33]. Moreover, IFN-γ enhances the expression of genes involved in innate immunity, thus contributing further to the mechanisms inhibiting *Chlamydia* infections [34]. Our findings revealed that FP can significantly induce IFN-γ not only at the transcription level but also at the protein level, in contrast to BUD, indicating the previously described phenomenon that FP preserves IFN-γ responses [29]. IFN-γ associated MIG/CXCL9 was shown to have an antimicrobial effect to *C. pneumoniae* [35], similar to *C. trachomatis* and *C. muridarum* [34]. Our results demonstrated that MIG/CXCL9 levels increased at the protein level in parallel with IFN-γ after FP administration, compared to that in untreated control and BUD-treated mouse lungs. Interestingly, FP did not alter the expression of other IFN-γ-inducible chemokines, suggesting that other mechanisms may be playing a role in chemokine expression. Notably, BUD treatment significantly reduced the gene expression levels of CXCL-10 and CXCL-11, which is in accordance with a previous report [36]. Consistently, ICS treatments have a unique impact on IFN-γ response and chemokine production.

IDO activity is a hallmark of tryptophan depletion and suppression of *Chlamydia* growth in cell cultures and mice. Furthermore, IDO activity is associated with several pulmonary diseases, including lung cancer [37]. Sputum IDO activity was enhanced in an IL-10 dependent manner in asthmatic patients receiving ICSs; the ICSs increased IL-10 secretion from macrophages in parallel with IDO activity, indicating that ICS use could generate IDO activity through IL-10 production [38]. Our results also indicate a significant increase in IL-10 production after FP administration in infected mice; conversely, BUD did not have an enhancing effect. Since it is well-known that FP can increase local secretion of IL-10 in vivo [39], we concluded that the observed IDO activity could have been derived from the additive effects of IFN-γ and IL-10. 

To further analyse the production of anti-chlamydial cytokines, we measured IL-17A levels, as it has been reported to trigger neutrophil recruitment in the lung, whereas its neutralisation results in a higher *C. pneumoniae* burden [23]. FP was not able to inhibit IL-17A, in contrast to BUD, which downregulates IL-23, the potential inducer of IL-17A [40,41]. Our current findings revealed that FP-treated mice produced significantly higher amounts of IL-17A in response to *C. pneumoniae* infection, suggesting that this increase in IL-17A production might also result in the development of *C. pneumoniae* inhibition; as observed in a previous study, IL-17A can synergise with IFN-γ, playing a protective role in *Chlamydia* infection [42].

Lastly, we analysed the relative expression of GR and VDR, and we found that FP treatment significantly increased VDR expression compared to control and BUD-treated mice. VDR is one of the highly downregulated transcription factors in *Chlamydia*-infected murine cells [34], suggesting that the elevated VDR expression in our study was due to the influence of FP in mice. This result raises the likelihood of the beneficial impact of FP to stem from VDR activation; a former study proved that the diminished VDR activity was associated with higher *Chlamydia* load in the lungs [43]. Vitamin D favours the curtailment of bacterial infections, and it had several implications in modulating adaptive and innate immunity to eliminate bacterial infections [44]. Therefore, our results raise the possibility that FP supports VDR functions, which can further influence cytokine and chemokine production. Thus, we recommend further examination of the interaction between ICS use and VDR activity, as Vitamin D supplementation is widely recommended in asthma.

In addition, we investigated whether FP or BUD could amend the histopathology of *C. pneumoniae*-infected mouse lungs and found that all specimens contained lymphocyte infiltration as a sign of *C. pneumoniae* infection. However, emphysema was also visible in FP-treated lung samples. As FP treatment showed an exaggerated IFN-γ response to the *Chlamydia* infection, the elimination of *C. pneumoniae* through enhanced cytokine and chemokine production could lead to minimal emphysema in infected lungs. According to previous studies [45,46], emphysema can be associated with the IFN-γ production and *Chlamydia* infections.

To our knowledge, this is the first study that investigates the effects of FP and BUD on *C. pneumoniae* infection in vivo and in vitro. The most notable results of our research are as follows: (1) FP treatment inhibited *C. pneumoniae* growth in A549 cells; (2) FP also inhibited *C. pneumoniae* replication in vivo; (3) FP induced IFN-γ at the gene expression and protein levels, leading to enhanced IDO activity and MIG/CXCL9 production; (4) FP promoted the expression of VDR.

## 4. Materials and Methods

### 4.1. In Vitro Study Design

A549 human airway epithelial cells (ATCC, Manassas, VA, USA) were transferred to a 96-well plate at a density of 4 × 10^4^ cells/well in 100 μL of minimal essential medium (MEM) with Earle’s salts supplemented with 10% heat-inactivated foetal bovine serum (FBS), 2 mmol/L-glutamine, 1x non-essential amino acids, 4 mM HEPES and 25 μg/mL gentamycin. The cells were pre-treated and incubated for 24 h at 37 °C, 5% CO_2_ with FP (Sigma-Aldrich, Saint Louis, MO, USA) or BUD (Sigma-Aldrich, Saint Louis, MO, USA) or left untreated. The highest non-toxic drug concentrations (FP: 3.5 × 10^−4^ mM, BUD: 7 × 10^−4^ mM) determined by the 3-(4,5-dimethylthiazol -2yl)-2,5-diphenyl-2H-tetrazolium bromide (MTT) cytotoxicity test. After 24 h treatment, the wells were washed twice with phosphate-buffered saline (PBS) and the cells were infected with *C. pneumoniae* at a multiplicity of infection (MOI) of 0.01. The cells were inoculated in 0.5% (*w*/*v*) glucose medium and centrifuged (800× *g*, 60 min), which was followed by the addition of FP or BUD to the wells. Control-infected cells were left untreated. After infection, the plates were incubated at 37 °C, under 5% CO_2_ for 48 h. Subsequently, the wells were washed twice with PBS, and 100 μL sucrose–phosphate–glutamic acid (SPG) solution was added to each well. The plates were subjected to two freeze–thaw cycles with a quick freezing (−80 °C, 15 min) to obtain cell lysates, which were used directly as templates for quantitative polymerase chain reaction (qPCR). To evaluate *C. pneumoniae* propagation, direct qPCR was performed as described previously [18,47].

### 4.2. Inoculum Preparation and Immunostaining

*C. pneumoniae* strain CWL-029, kindly gifted by Agathe Subtil (Pasteur Institute, Paris, France), was propagated on HEp-2 cells as described previously [48]. The elementary bodies (EBs) were purified and concentrated, and subsequently aliquoted in SPG, which was followed by storage at −80 °C until further use. Indirect immunofluorescence was performed to determine the concentration of infectious *C. pneumoniae* EBs. Serial dilutions of purified EBs were inoculated onto McCoy cell monolayers (ECACC, London UK). After incubation for 48 h, the infected cells were fixed with acetone at −20 °C and stained with monoclonal anti-*Chlamydia* LPS antibody (AbD Serotec, Oxford, UK) and fluorescein isothiocyanate (FITC)-labelled anti-mouse immunoglobulin (Ig) G (Sigma-Aldrich, St. Louis, MO, USA). The number of *C. pneumoniae* inclusions was counted under a UV microscope, and the titre was expressed as inclusion-forming units (IFUs)/mL.

### 4.3. Corticosteroid Treatment in Mice

FP and BUD powder were obtained, and dimethyl sulfoxide (DMSO) was used as a vehicle for the drugs. Mice were exposed to nebulised BUD (40 μg, 1000 μg/kg) and FP (25 μg, 625 μg/kg) in an inhalation chamber for 15 min once a day, as described previously [49]. We used BUD and FP at equivalent concentrations with the ratio FP:BUD = 1:1.6, based on former clinical studies [50] and the higher potency of FP [10].

### 4.4. Animals and Experimental Design

Female BALB/c mice (6–8-weeks-old) were obtained from Charles River Laboratories (Hungary). The mice were kept under standard husbandry conditions at the animal facility of the Department of Medical Microbiology and Immunobiology, University of Szeged. Animals were fed regular mouse chow and provided with water *ad libitum*. The mice were randomly divided into three groups: the control, the BUD-treated, and FP-treated (n = 16 in each group). Mice received either BUD, FP, or vehicle alone, for three days prior to infection, and then for seven days after infection. On day 3, the mice were sedated with intraperitoneal injection of sodium pentobarbital (200 μL, 7.5 mg/mL) and were infected with 5 × 10^5^ IFU *C. pneumoniae* in 20 μL SPG. On day 10, i.e., seven days after infection, the mice were anaesthetised and sacrificed. The lungs were removed and homogenised with acid-purified sea sand (Fluka Chemie AG, Buchs, Switzerland) using a mortar with a pestle. One half of the homogenised lungs was prepared for total RNA extraction, and the other half was suspended in 1 mL SPG for the detection of recoverable *C. pneumoniae* and for cytokine measurements. The experiments were implemented with the approval of the Animal Welfare Committee of the University of Szeged, Hungary and conformed to the Directive 2010/63/EU.

### 4.5. Culturing of C. pneumoniae from the Lungs

One half of the homogenised lungs was centrifuged (10 min, 400× *g*), and serial dilutions of the supernatants were inoculated onto McCoy cell monolayers and centrifuged (60 min, 800× *g*). The number of recoverable *C. pneumoniae* inclusions was determined by indirect immunofluorescence as described earlier, and expressed in terms of IFU/mL.

### 4.6. mRNA Extraction and cDNA Synthesis

Total RNA was extracted from the other half of the homogenised lung tissues of the control (n = 12), as well as BUD- and FP-treated mice (n = 12 for each group) using TRI reagent (Sigma-Aldrich, Saint Louis, MO, USA) according to the manufacturer’s protocol. Total RNA concentrations and purity were measured using a NanoDrop spectrophotometer (Thermo Scientific, Waltham, MA, USA). First-strand cDNA was synthesised from 2 μg of total RNA using Maxima First Strand cDNA Synthesis Kit, and 20 pM random hexamer primer in 20 μL reaction buffer according to the manufacturer’s protocol (Thermo Fisher Scientific Inc. Waltham, MA, USA).

### 4.7. qPCR Validation

qPCR was performed in a Bio-Rad CFX96 real-time system with SsoFast™ EvaGreen® qPCR Supermix (Bio-Rad, Hercules, CA, USA) master mix and murine specific primer pairs: β-actin sense, 5′-TGGAATCCTGTGGCATCCATGAAAC-3′; β-actin antisense, 5′-TAAAACGCAGCTCAGTAACAGTCCG-3′; IDO1 sense, 5′-GCTTCTTCCTCGTCTCTCTATTG-3′; IDO1 antisense, 5′-TCTCCAGACTGGTAGCTATGT-3′; IDO2 sense, 5′-CCTGGACTGCAGATTCCTAAAG-3′; IDO2 antisense, 5′-CCAAGTTCCTGGATACCTCAAC-3′; TDO sense, 5′-GGCATGGCTGGAAAGAACAC-3′; TDO antisense, 5′-CTCCCTGGAGTGCACGGTAT-3′; IFN-γ sense, 5′-CAAGTGGCATAGATGTGGAAGA-3′; IFN-γ antisense, 5′-GCTGTTGCTGAAGAAGGTAGTA-3′; MIG/CXCL9 sense, 5′-ACGTAGGTTTCGAGACCAGGGATT-3′; MIG/CXCL9 antisense, 5′-CAACACCAAGTGTTCTGCCACCAA-3′; IP10/CXCL10 sense, 5′-TGGCTAGTCCTAATTGCCCTTGGT-3′; IP10/CXCL10 antisense, 5′-TCAGGACCATGGCTTGACCATCAT-3′; ITAC/CXCL11 sense, 5′-TACCCGAGTAACAGCTGCGACAAA-3′; ITAC/CXCL11 antisense, 5′-TATGAGGCGAGCTTGCTTGGATCT-3′; VDR sense, 5′-TACACCCCCTCACTGGACATGAT-3′; VDR antisense, 5′-CGATGACCTTTTGGATGCTGTAA-3′; GR sense, 5′-GTTCCTAAGGAAGGTCTGAAGAG-3′; and GR antisense, 5′-CAATTCTGACTGGAGTTTCC-3′.

Cycle threshold (Ct) values were calculated for β-actin, IDO1, IDO2, TDO, IFN-γ MIG/CXCL9, IP10/CXCL10, ITAC/CXCL11, VDR, and GR, and the relative gene expression levels were determined by the 2^−(ΔΔCt)^ method. The relative expression level was indicated as 2^−(ΔΔCt)^, where ΔΔCt = ΔCt for the experimental sample −ΔCt for the control sample.

### 4.8. Lung Histology

Microscopic examination of the lungs of infected control as well as BUD-treated and FP-treated mice (n = 4 from each group) was performed. After the removal of the lungs, tissues of individual mice were immediately placed into plastic tubes, pre-filled with 10% formalin, resulting in 1:10 of tissue/formalin ratio. During dissection, tissue samples in the tube were cut into 1 mm slices and embedded into paraffin blocks. Four-micrometre sections were cut, and regular haematoxylin–eosin (HE) staining was performed. All tissue fragments were examined by light microscopy.

### 4.9. Cytokine and Chemokine Measurements from the Lungs

The supernatants of homogenised lung tissues were centrifuged (12,000× *g*, 5 min) and enzyme-linked immunosorbent assay (ELISA) for IFN-γ, IL-4, IL-10, IL17-A, MIG/CXCL-9 was performed according to the manufacturers’ instructions. MIG/CXCL-9 concentration was determined using a mouse MIG/CXCL-9 ELISA Kit (Sigma-Aldrich, Saint Louis, MO, USA) and IL-17A was measured using Quantikine mouse IL-17 immunoassay (R&D Systems, Minneapolis, MN, USA). IFN-γ, IL-4, and IL-10 concentrations were detected with Invitrogen mouse ELISA kits (Thermo Fisher Scientific Inc., Waltham, MA, USA). Sensitivity for IFN-γ, IL-4, IL-10, IL-17A, and MIG/CXCL-9 measurement ranges were 15–2000, 4–500, 32–4000, 10.9–700, and 2.741–2000 pg/mL, respectively.

### 4.10. Statistical Analysis

Statistical analysis of data was performed with GraphPad Prism 8.0.1. software, using one-way and two-way ANOVA, and Kruskal–Wallis test. All post hoc comparisons were performed using Tukey’s method. Data are expressed as mean ± standard deviation (SD). Differences at *p* < 0.05 were considered statistically significant.

## 5. Conclusions

Our results demonstrate that FP treatment can lead to favourable outcomes in *C. pneumoniae* infection by enhancing IFN-γ responses, and inducing potential anti-chlamydial cytokine and chemokine production. Our observations revealed that the effects of FP were different from other ICSs; therefore, we hypothesised that FP is beneficial in *C. pneumoniae* infections. Recently, patients with asthma are controlled in new ways via telemedicine and experienced fewer outpatient visits due to COVID-19 pandemic. Therefore, investigating the associations of ICSs use and respiratory infections is especially important to avoid further asthma exacerbations. Our study can contribute to a better management of asthma and *C. pneumoniae* infections, and it could assist therapeutic choices.

## Data Availability

The data presented in this study are available on request from the corresponding author. The data are not publicly available due to privacy.
